# Photoreceptor protection via blockade of BET epigenetic readers in a murine model of inherited retinal degeneration

**DOI:** 10.1186/s12974-016-0775-4

**Published:** 2017-01-19

**Authors:** Lei Zhao, Jun Li, Yingmei Fu, Mengxue Zhang, Bowen Wang, Jonathan Ouellette, Pawan K. Shahi, Bikash R. Pattnaik, Jyoti J. Watters, Wai T. Wong, Lian-Wang Guo

**Affiliations:** 1 Department of Surgery, 5151 Wisconsin Institute for Medical Research, University of Wisconsin School of Medicine and Public Health, Madison, WI 53705 USA; 2 Department of Ophthalmology, The First Hospital of China Medical University, Shenyang, 110001 People’s Republic of China; 3 Department of Ophthalmology, The 3rd People’s Hospital of Dalian, Dalian, 116033 People’s Republic of China; 4 Shanghai Key Laboratory of Psychotic Disorders, Shanghai Mental Health Center, Shanghai Jiao Tong University School of Medicine, 600 Wanping Nan Road, Shanghai, 200030 People’s Republic of China; 5 Department of Comparative Biosciences, University of Wisconsin, Madison, WI 53706 USA; 6 Department of Pediatrics, University of Wisconsin, Madison, WI USA; 7 Unit on Neuron-Glia Interactions in Retinal Disease, National Eye Institute, National Institutes of Health, Bethesda, MD USA; 8 McPherson Eye Research Institute, University of Wisconsin, Madison, WI 53705 USA; 9 Department of Ophthalmology and Visual Sciences, University of Wisconsin, Madison, WI USA

**Keywords:** Bromodomain and extraterminal domain (BET) proteins, Epigenetic readers, Retinal degeneration, Microglial activation, JQ1, rd10 mice

## Abstract

**Background:**

The bromodomain and extraterminal domain (BET) family proteins (BET2, BET3, and BET4) “read” (bind) histone acetylation marks via two distinct bromodomains (Brom1 and Brom2) facilitating transcriptional activation. These epigenetic “readers” play crucial roles in pathogenic processes such as inflammation. The role of BETs in influencing the degenerative process in the retina is however unknown.

**Methods:**

We employed the rd10 mouse model (*Pde6b* ^*rd10*^ mutation) of retinitis pigmentosa (RP) to examine the involvement of BET proteins in retinal neurodegeneration.

**Results:**

Inhibition of BET activity by intravitreal delivery of JQ1, a BET-specific inhibitor binding both Brom1 and Brom2, ameliorated photoreceptor degeneration and improved electroretinographic function. Rescue effects of JQ1 were related to the suppression of retinal microglial activation in vivo, as determined by decreased immunostaining of activation markers (IBA1, CD68, TSPO) and messenger RNA (mRNA) levels of inflammatory cytokines in microglia purified from rd10 retinas. JQ1 pre-treatment also suppressed microglial activation in vitro, decreasing microglial proliferation, migration, and mRNA expression of inflammatory cytokines (TNFα, MCP-1, IL-1β, IL-6, and RANTES). Expression of BET2, but not BET3 and BET4, was significantly elevated during photoreceptor degeneration at postnatal day (PN)24 in retinas of rd10 mice relative to age-matched wild-type controls. siRNA knockdown of BET2 but not BET4, and the inhibitor of Brom2 (RVX208) but not of Brom1 (Olinone), decreased microglial activation.

**Conclusions:**

These findings indicate that BET inhibition rescues photoreceptor degeneration likely via the suppression of microglial activation and implicates BET interference as a potential therapeutic strategy for the treatment of degenerative retinal diseases.

**Electronic supplementary material:**

The online version of this article (doi:10.1186/s12974-016-0775-4) contains supplementary material, which is available to authorized users.

## Background

Retinitis pigmentosa (RP) is a major blinding disease characterized by photoreceptor degeneration arising predominantly from mutations in genes expressed in photoreceptor or RPE cells [1, 2]. Despite extensive studies and trials of various means, e.g., antioxidants and stem cell therapy, to preserve or replace photoreceptors in RP, few effective clinical treatments are currently available [2]. Recently, gene correction or gene therapy has shown promise to treat RP [1, 3]. However, the significant number (>170) of RP-causative genes [4] is a sobering reminder that it is imperative to identify and target a common mechanism or regulator shared by various RP etiologies.

Neuroinflammation is now considered a hallmark of many neurodegenerative disorders [5]. Hyper-activation of microglia, a class of innate immune cells, was recently demonstrated to be an important contributor to photoreceptor neurodegeneration in the rd10 (*Pde6b*) model of RP [6]. Most recently, a report using the rd10 model discovered a positive feedback mechanism whereby activated microglia migrate to and phagocytose non-apoptotic photo-receptors and then become even more activated, profoundly accelerating the loss of both non-apoptotic and apoptotic photoreceptors [7]. Significantly, pathogenic microglial activation is associated with photoreceptor loss not only in RP but also age-related macular degeneration and diabetic retinopathy in animal models and human patients [8]. Thus, blocking microglial over-activation emerges as an appealing strategy to improve photoreceptor survival across various etiologies of retinal degeneration. However, poor understanding of the molecular mechanism(s) underlying microglial activation, particularly in the retina, poses a major barrier to applying this strategy [8].

Recent groundbreaking studies suggest that the bromodomain and extraterminal domain (BET) family of epigenetic “readers” is a powerful regulator in pathogenesis involving inflammation [9–11]. For BET family proteins, hereafter referred to as BET2, BET3, and BET4 (BRDs in the literature) [12], each contains two distinct bromodomains (denoted as Brom1 and Brom2 in this report) and an extraterminal domain. They “read,” i.e., recognize and bind, acetylation marks on histones and/or on transcription factors via their bromodomains and “translate” the chromatin marking into gene expression by activating transcriptional machinery [12]. The BET family was widely viewed as undruggable, until the serendipitous discovery of the first-in-class inhibitor JQ1 [13], and subsequently its derivatives that specifically block BET bromodomains [14]. Importantly, BET bromodomain blockade effectively mitigates cancers and inflammatory diseases. Several BET inhibitors have quickly entered clinical trials and shown encouraging results [14]. Of particular relevance to the current study, BET inhibitors abrogate the activation of macrophages [9, 15]. These adaptive immune cells share many characteristics with microglia [16], raising a question as to whether the BET family also plays a role in microglial activation. In support of this, a new report shows that JQ1 mitigates the expression of inflammatory cytokines in the BV-2 microglial cell line [17]. However, the specific roles of BET proteins and their bromodomains in the activation of microglia in the retina, and in RP, are not known.

The current report is the first to address the role of the BET family in retinal degenerative disease. We asked whether blocking the BET family with JQ1 promotes photoreceptor survival in a well-established RP model (rd10 mice). We then determined the JQ1 effect on microglial activation, as this pathogenic process is known to greatly exacerbate photoreceptor loss in rd10 mice [6, 7]. Our data indicate that JQ1 treatment abrogates microglial activation in vitro, and in vivo in the rd10 retina, and also effectively preserves rd10 mouse photoreceptor cell survival and function. These results implicate a new paradigm for RP treatment by targeting BET epigenetic readers.

## Methods

### Animals

All animal procedures conformed to the NIH guide for the ethical care and use of laboratory animals and were in compliance with the ARVO Statement for the Use of Animals in Ophthalmic and Vision Research. Animal protocols were approved by the Institutional Animal Care and Use Committee of the University of Wisconsin-Madison. All surgeries were performed under isoflurane anesthesia (inhalation at 2 ml/min flow rate), and every effort was made to minimize animal suffering. Animals were euthanized in a chamber gradually filled with CO_2_. Wild-type (WT; C57BL/6) and rd10 mice were purchased from the Jackson Laboratory (Bar Harbor, ME). Animals were maintained on a 4% fat diet (8604 M/R, Harkland Teklad, Madison, WI) and subjected to standard light cycles (12 h/12 h light/dark).

### Intravitreal injection of JQ1 in rd10 mice

To test the effect of BET bromodomain blockade in vivo, 2 μl of JQ1 (0.1 mM dissolved in 10% DMSO in PBS) or vehicle (10% DMSO in PBS) was intravitreally injected into rd10 or WT mice at PN14. WT mice were only injected with vehicle. Each rd10 mouse received JQ1 in one eye (left or right, randomly assigned) and vehicle in the contralateral control eye. At time points indicated in figures, animals were either subjected to ERG measurements or euthanized for preparation of retinal homogenates or PFA-fixed sections. Animals that developed complications from the injection procedure (e.g., ocular infection, inflammation) were excluded from the analysis. This criterion was pre-established and involved <5% of treated animals. The experiments were performed independently at least three times.

### Electroretinogram recordings

Ten days after intravitreal injection (PN24) of vehicle or JQ1 in rd10 mice, ISCEV standard full-field flash ERG was performed using HMsERG system (OcuScience, Henderson, NV) following our published method [18]. Mice were dark-adapted overnight and anesthetized with intraperitoneal ketamine (90 mg/kg) and xylazine (8 mg/kg) under dim-red illumination. After topical application of tropicamide (1%, Alcon) and phenylephrine (2.5%, Alcon) for pupillary dilation and proparacaine hydrochloride (0.5%, Alcon) for topical anesthesia, stainless steel subdermal needle electrodes were placed for ground (at the tail) and under individual eye lids as reference electrodes. Rodent 2.5-mm contact lens with silver-embedded thread electrode were placed on the cornea of each eye using Goniovisc hypromellose 2.5% ophthalmic lubricant solution (HUB Pharmaceuticals, CA). Flash ERG recordings were obtained simultaneously from both eyes at increasing light intensities from 0.03 to 30 cd s/m^2^ (saturating intensity in our reported studies [18]) under dark-adapted conditions. The stimulus interval between flashes varied from 20 s at the lowest stimulus strengths to 60 s at the highest ones. Two to 10 responses were averaged depending on flash intensity. ERG signals were sampled at 1 kHz and recorded with 0.3 Hz low-frequency and 300 Hz high-frequency cutoffs. Analysis of a-wave and b-wave amplitudes was performed using ERGView analytical software (OcuScience, Henderson, NV) that digitally filters out high-frequency oscillatory potential wavelets. The a-wave amplitude was measured from the baseline to the negative peak, and the b-wave was measured from the a-wave trough to the maximum positive peak and plotted using Origin.

### Preparation of retinal sections and homogenates

At various time points after injection (PN14, 18, 21, 24, 30), animals were euthanized by CO_2_ asphyxiation followed by cervical dislocation. Eyeballs were immediately enucleated and dissected. For morphometric and immunohistochemistry analyses, eyeballs were fixed in 4% paraformaldehyde for 7 h at 4 °C, and then used for preparation of paraffin-embedded sections or cryosections, according to our published methods [19]. Briefly, for cryosections, eyeballs were soaked in 30% sucrose in PBS for 14 h at 4 °C and 10-μm sections were cut from the eyeballs frozen in an optimum cutting temperature (OCT) embedding medium (Sakura Finetek 4583; Sakura Finetek USA, Inc., Torrance, CA). For paraffin-embedded sections, eyeballs were dehydrated by ethanol/xylene after fixation and embedded in paraffin. Ten-micrometer-thick sections were prepared for immunohistochemistry. For Western blotting and quantitative real-time PCR (qRT-PCR) determination, tissue homogenates were prepared from unfixed retinas.

### Photoreceptor counting in the outer nuclear layer

The number of photoreceptors on retinal paraffin sections was evaluated by counting H&E-stained nuclei in the outer nuclear layer (ONL) following our published method with minor modifications [7]. Briefly, for each section, the central, middle, and peripheral regions were defined as 0–1000 μm, 1000–2000 μm, and greater than 2000 μm from the optic nerve head, respectively. Nuclei were manually counted in each (100 μm length of retina) of the three fields chosen in the central, middle, and peripheral regions of the ONL. The counts from all three to four sections of the same eye were averaged, and the means from six to nine animals were then averaged to calculate the mean and standard error of the mean (SEM) for each group of animals.

### Immunohistochemistry for assessment of protein levels of BETs in the retina

Immunostaining was performed on paraffin-embedded retinal sections following our published method [19]. Briefly, sections were first incubated with each of the primary antibodies for 1 h: rabbit anti-BET2 (1:150, Abcam, 139690, Cambridge, MA); rabbit anti-BET4 (1:200, Abcam, 128874); mouse anti-BET3 (1:200, Abcam, 56342). Sections were then incubated with ImmPRESS HRP-conjugated goat-anti-rabbit (or mouse) secondary antibody (1:200, Vector Laboratories), followed by visualization with 3,3′-diaminobenzidine (DAB) and counterstaining with hematoxylin. Sections stained with the secondary antibody, but not a primary antibody, were used as a background control.

### Immunohistochemistry and fluorescence microscopy for assessment of retinal microglial distribution

Immunostaining was performed on retinal cryosections following our previously described method [20] with minor modifications. Briefly, retinal sections were permeabilized with 1% Triton X-100 in PBS for 20 min, blocked with 10% normal donkey serum (017-000-121; Jackson Immunoresearch Lab, MS) for 2 h at room temperature, and then incubated with a primary antibody overnight at 4 °C. Sources and dilutions of primary antibodies are the following: rabbit anti-IBA-1 (Waco, 019-19741), 1:400; rabbit anti-CD68 (Millipore, MAB3402), 1:200; rabbit anti-TSPO (Abcam, 109497, Cambridge, MA), 1:200. After rinsing the section three times, a secondary antibody (Alexa-488 conjugated donkey-anti-rabbit or Alexa-594-conjugated donkey-anti-mouse) at 2 μg/ml was applied at room temperature for 2 h. Sections were then rinsed three times, counterstained with 4′,6-diamidino-2-phenylindole (DAPI) for 5 min, and then mounted in Prolong Gold mounting medium (Invitrogen, Carlsbad, CA) and cover-slipped. The slides were left in the dark overnight and then sealed using clear nail polish (Electron Microscopy Sciences, Hatfield, PA). Images were acquired with a Nikon Eclipse Ti microscope with a DS-Qi1 camera using ×20 or ×40 objective lens and analyzed by Nikon Elements software. Immuno-fluorescence from the central, middle, and peripheral regions was quantified manually. Cell counts from all three to four sections of the same eye were averaged, and the means from six to nine animals were then averaged to calculate the mean and SEM for each group of animals. Sections stained with a secondary antibody, but not a primary antibody, were used as background control.

### TUNEL labeling and caspase-3/7 activity assay

Terminal deoxynucleotidyl transferase dUTP nick end labeling (TUNEL) assay was performed using an In Situ Cell Death Detection Fluorescein or TMR red kit (Roche, Indianapolis, IN, USA). The TMR red kit was used for co-staining of other markers. TUNEL staining was performed on retinal cryosections and imaged to assess DNA fragmentation as an indicator of apoptosis. TUNEL-positive cells were quantified in three fields from three to four sections per eye. Each field represented a 500-μm retinal length in the central, middle, and peripheral regions. Cells were scored as either positive or negative. The counts from all sections of the same animal were averaged for a per animal mean, and the means from six to nine animals were averaged to generate the mean and SEM for the entire group of animals.

A Caspase-Glo 3/7 assay kit (Promega, Madison, WI) was used to determine the relative activity of caspase-3/7 according to manufacturer’s instructions. Briefly, in a 96-well plate, retinal homogenates were incubated with 50 μl Caspase-Glo 3/7 reagent and 50 μl PBS (per well). Plates were incubated at room temperature for 1 h and read in a FlexStation 3 Benchtop Multi-Mode Microplate Reader (Molecular Devices, Sunnyvale, CA).

### Microglia isolation and purification from rd10 mouse retinas

JQ1 or vehicle was intravitreally injected to rd10 mice at PN14, as described above. After 10 days, retinal microglial cells were isolated and purified, following our published method with minor modifications [7]. Briefly, animals were euthanized and their eyeballs enucleated immediately. The globes were dissected free of periocular connective tissues and rinsed with HBSS buffer. The anterior segment and vitreous were removed, and the retina was dissected free from the underlying RPE layer. The retinas were transferred into DMEM containing 70 U/ml collagenase (0.5 ml per eye) and incubated at 37 °C for 60 min. Enzyme activity was terminated using DMEM containing 10% FBS. The retinas were dissociated mechanically and passed through 40-μm nylon mesh (Corning, NY). The dissociated cells were then labeled with antibodies for CD11b (BD, 557397) and CD45 (BD, 559864) and DAPI. Microglial cells were purified by flow sorting (CD11b positive and CD45 low) and a >95% purity was achieved. Cells were used immediately for RNA isolation and qRT-PCR.

### Microglia isolation and purification from B6 mouse brains

Primary microglial cells were prepared as we described previously [21]. Briefly, brains from 3–5-day-old mice were dissected and dissociated with 0.25% trypsin supplemented with EDTA followed by trituration with a Pasteur pipette until a single cell suspension was obtained. Cells were resuspended in DMEM supplemented with 10% FBS and 100 units/ml penicillin/streptomycin and plated in 80-mm^2^ cell culture flasks. After 10–12 days, flasks were gently shaken for 1 h and the medium was harvested and centrifuged for 10 min to collect microglia.

### Real-time quantitative PCR assay for expression of inflammatory cytokines

RNA was extracted from retinal homogenates or cells using Trizol (QIAGEN, Valencia, CA) following the manufacturer’s instructions. Purified messenger RNA (mRNA) (1 μg) was used for the first-strand complementary DNA (cDNA) synthesis using iScript cDNA synthesis kit (Bio-Rad) and quantitative RT-PCR was performed using the 7500 Fast Real-Time PCR System (Applied Biosystems, Carlsbad, CA), as described in our previous report [22]. Real-time quantitative PCR (qRT-PCR) data with a high cycle number (e.g., >35) was not considered. Each cDNA template was amplified in triplicate using SYBR Green PCR Master Mix (Applied Biosystems, Carlsbad, CA).

### Enzyme-linked immunosorbent assay for MCP-1 protein production

ELISA was performed to evaluate MCP-1 protein production in microglial cells, using an MCP-1 ELISA kit based on the sandwich enzyme immunoassay technique (R&D Systems, Minneapolis, MN, USA). The absorbance was determined using a Flex Station 3 microplate reader (Molecular Devices, Sunnyvale, CA, USA).

### N9 microglial cell culture, pre-treatment with BET inhibitors, and LPS stimulation

Mouse N9 microglial cells were kindly provided by Dr. Paula Ricciardi-Castagnoli [23] and grown in the same medium described above for primary microglial cells. Cells were plated at a density of 120,000 cells/well on 12-well plates and used for experiments the following day. (+)-JQ1 (Cayman Chemicals, Ann Arbor, MI, USA), Olinone (Cat. GLXC-05021, Glixx Laboratories, Southborough, MA, USA), and RVX208 (Apexbio, Houston, TX, USA) were dissolved in dimethyl sulfoxide (DMSO, Sigma-Aldrich, St. Louis, MO, USA) for preparation of stock solutions, which were then diluted in DMEM for experiments. The final concentration of DMSO in the medium was less than 10 μL/10 mL, which did not show any effect on cell growth. To identify appropriate concentrations of BET inhibitors for various experiments, we performed dose response pilot studies. N9 cells were pre-treated with BET inhibitors at various concentrations for 12 h, and then subjected to CellTiterGlo viability assay, as described in our previous report [22]. We chose 0.5, 30, and 30 μM for JQ1, Olinone, and RVX208, respectively, as these represent the maximal concentrations that did not compromise N9 cell viability (Additional file 1: Figure S6). For experiments to evaluate the effect of BET inhibitors on lipopolysaccharide (LPS)-stimulated phenotypes of activated microglia (N9 or primary cells), cells were pre-treated with JQ1, Olinone, or RVX208 for 12 h and then stimulated with LPS (1 μg/mL, Sigma-Aldrich, St. Louis, MO, USA) for 2 h followed by various assays as described below in detail.

### N9 microglia cell proliferation assay (BrdU)

To study the effect of BET inhibitors on the proliferation of N9 microglial cells, we used a Cell Proliferation BrdU ELISA (colorimetric) Kit (Roche Applied Science, Indianapolis, IN) following manufacturer instructions, as described in our previous study [22] with minor modifications. Briefly, N9 cells were seeded in 96-well plates at a density of 4000 cells per well with a final volume of 200 μl, in DMEM containing 0.5% FBS. Cells were pre-treated with 0.5 μM JQ1, 30 μM Olinone, 30 μM RVX208, or an equal volume of vehicle control (DMSO) for 12 h prior to LPS stimulation (final 1 μg/ml). After LPS treatment for 2 h, cells were labeled with BrdU in DMEM containing 10% FBS for a 2-h incubation at 37 °C, and then fixed with a FixDenat solution for 30 min, followed by a 90-min incubation at room temperature with an anti-BrdU-POD antibody (1:100 dilution). After washing with PBS three times, substrate was added. Plates were incubated at room temperature for 30 min, and colorimetric signals were measured on a FlexStation 3 Benchtop Multi-Mode Microplate Reader (Molecular Devices, Sunnyvale, CA) at 370 nm with a reference wavelength of 492 nm.

### N9 microglia cell migration assay (Transwell)

Assay was performed according to our previously reported method [22]. Briefly, N9 cells were seeded at a density of 20,000/well in the upper chamber of Transwell Permeable Supports (or Inserts) (8 μm pore size, Corning, NY) placed in 24-well plates. Cells were pre-incubated with 0.5 μM JQ1, 30 μM Olinone, and 30 μM RVX208 or vehicle (DMSO) for 12 h prior to LPS stimulation (final 1 μg/ml). Inserts were harvested at 24-h post stimulation and fixed in 70% ethanol at −20 °C for 30 min. Pre-moistened cotton swabs were used to gently scrape remaining cells in the upper chamber of inserts, followed by staining the cells on the lower surface of the insert in hematoxylin solution for 30 min at room temperature. The upper chamber of the inserts was swabbed again and rinsed twice with PBS. After air-drying the inserts for 30 min, the polyester membranes were harvested using a scalpel and mounted on glass slides using 90% glycerol. Images were then taken to quantify cells that migrated across the membrane from the upper chamber to the lower surface.

### siRNA knockdown of BET proteins

Knockdown was performed as described in our previous report [22]. Lentiviruses for expression of scrambled or mouse BET-specific siRNAs were packaged using a three-plasmid expression system including piLenti-siRNA-GFP, psPAX2, and pMD2.G (Addgene, Cambridge, MA). The piLenti-siRNA-GFP vectors for expression of a scrambled siRNA or siRNAs specific for mouse BET2 or BET4 were purchased from Applied Biological Materials Inc. (Canada).

For BET2 knockdown, two siRNAs were used as a mixture:

5′-CCACAATGGCTTCTGTACCAGCTTTACAA-3′

5′-CCACAATGGCTTCTGTACCAGCTTTACAA-3′

For BET4 knockdown, four siRNAs were used as a mixture:

5′-GTGGATGCCGTCAAGCTGAACCTCCCTGA-3′

5′-GGACTTCAACACTATGTTTACAAATTGTT-3′

5′-GGAGATGACATCGTCTTAATGGCAGAAGC-3′

5′-CCCAGGAATTTGGTGCTGATGTCCGATTG-3′

The three plasmids were co-transfected into HEK293T cells in DMEM medium containing 1% FBS using a JetPrime Polyplus-transfection reagent (Polyplus-transfection Inc., New York, NY) following the manufacturer’s protocol. After transfection for 24 h, the medium containing transfection reagents was replaced with fresh DMEM medium containing 1% FBS. The culture medium was collected after 24-h incubation and passed through a 0.45-μm filter (EMD Millipore, MA) and then concentrated and titrated using Lenti-X™ Concentrator and Lenti-X™ qRT-PCR Titration Kit (Clontech Laboratories, Inc., Mountain View, CA). The lentivirus preparation was then applied to the N9 microglial cell culture together with 8 μg/ml polybrene and incubated for 24 h. Infected (green fluorescent) cells were recovered in fresh DMEM medium containing 1% FBS for 3 days and subjected to flow sorting. Sorted cells were cultured for 2–3 days and then used in Western blotting or qRT-PCR assays.

### Western blotting for assessment of BET protein levels

Retinal homogenates or cells were solubilized in RIPA buffer containing protease inhibitors (50 mM Tris, 150 mM NaCl, 1% Nonidet P-40, 0.1% sodium dodecyl sulfate, and 10 μg/ml aprotinin). Protein concentrations of cell lysates were determined using a Bio-Rad DC™ Protein Assay kit. Approximately 15–30 μg of proteins from each sample was separated on 4–20% Mini-PROTEAN TGX precast gels (Bio-Rad) and transferred to PVDF membrane. Proteins of interest were detected by immunoblotting using the following primary antibodies and dilution ratios: rabbit anti-BET2 (1:1000) from Abcam (ab139690) and Bethyl laboratories (A302-583A), mouse anti-BET3 (1:1000) from Abcam (56342), rabbit anti-BET4 (1:1000) from Abcam (128874), and mouse anti-β-actin from Sigma-Aldrich. After incubation with HRP-conjugated secondary antibodies (1:5000, goat anti-rabbit or mouse, Bio-Rad), specific protein bands on the blots were visualized by applying enhanced chemiluminescence reagents according to the manufacturer’s instructions (Pierce, Rockford, IL) and then recorded with a LAS-4000 Mini imager (GE, Piscataway, NJ). Band intensity was quantified using ImageJ.

### Statistical analysis

The required sample sizes in animal experiments were calculated based on estimates of mean differences, variances, and power. Statistically significant differences between treatment groups were determined by one-way ANOVA (SPSS software, v.13.0, Chicago, IL) using the Bonferroni multiple comparison post hoc test or a two-tailed *t* test for grouped comparison. Significance was set at *P* < 0.05.

## Results

### Blocking BETs with JQ1 preserves photoreceptor number and function in rd10 mice

**Fig. 1:**
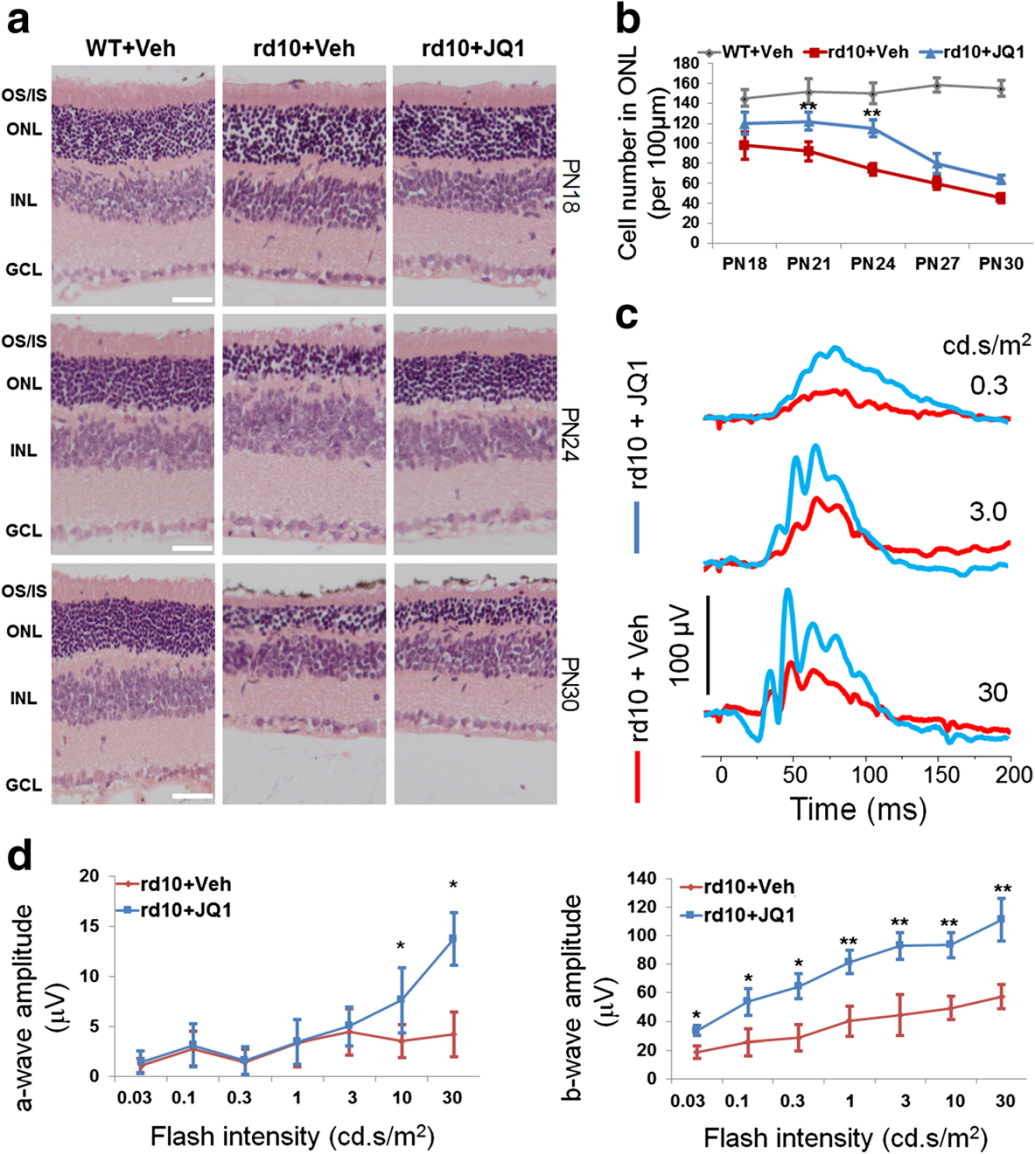
JQ1 treatment preserves photoreceptor number and function in rd10 mice. Vehicle (DMSO) and JQ1 (0.1 mM) were intravitreally injected, respectively, into one eye and the contralateral eye of rd10 mice at PN14. WT (B6) mice were injected only with vehicle. At PN18, PN21, PN24, PN27, and PN30, mice were either subjected to ERG measurements, or sacrificed for preparation of retinal sections. **a** Representative H&E-stained retinal paraffin sections showing a protective effect of JQ1 on ONL thickness in rd10 mice. *OS/IS* outer/inner segment, *ONL* outer nuclear layer, *INL* inner nuclear layer, *GCL* ganglion cell layer. *Scale bar* 50 μm. For images of PN21 and PN27, see Additional file 1: Figure S1. **b** Quantification for **a**: photoreceptor cell number per 100 μm ONL length; mean ± SEM, *n* = 6 mice; **P < 0.01 compared to rd10 vehicle control. **c** Representative ERG traces (measured at PN24, in response to 0.3, 3, or 30 cd s/m^2^ light intensity of flashes) showing rescue of rd10 mouse retinal function by JQ1 treatment. **d** Quantification of a-wave and b-wave amplitudes. ERG a-wave is the downward deflected negative response; b-wave is from the a-wave peak to positive response peak. Oscillatory potential is visible superimposed on b-wave. Data are presented as mean ± SEM, *n* = 10 mice, *P < 0.05, **P < 0.01 compared to rd10 vehicle control

The discovery of JQ1, the first-in-class inhibitor of the BET family [13], has opened unprecedented opportunities for studying the function of BET proteins in various diseases. While each BET protein contains two distinct bromodomains (Brom1 and Brom2), JQ1 binds to both in all three BETs [24]. JQ1 is a designer drug that is highly specific to the BET family as demonstrated in the studies against 46 non-BET bromodomains [13, 14, 25]. Thus, we used JQ1 to determine whether specifically blocking bromodomains of the BET family ameliorates photoreceptor loss. JQ1 and vehicle (DMSO) were injected, respectively, into the right (or left) eye and the contralateral eye of rd10 mice (PN14). Eyeballs were collected at PN18, PN21, PN24, PN27, and PN30 for the preparation of retinal sections, and photoreceptor numbers were counted in the outer nuclear layer (ONL) (Fig. 1a, Additional file 1: Figure S1). As shown in Fig. 1b, whereas photoreceptors continuously decreased from PN18 through PN30 in rd10 mice injected with vehicle, the photoreceptor number was at least 90% preserved until PN24 and then declined at later time points in rd10 mice injected with JQ1. The most prominent JQ1 protective effect was observed at PN24, the peak time of photoreceptor degeneration in rd10 mice [6].

We next determined the effect of JQ1 treatment on retinal function via electroretinogram (ERG) recording at PN24 (Fig. 1c). We found that the photoreceptor light response measured as the a-wave amplitude in vehicle-treated rd10 mice was completely obliterated at all flash intensities as expected for rd10 mice ERG responses, indicating loss of photoreceptor function. ERG a-wave amplitudes for JQ1-treated rd10 mouse retinas showed an increase in amplitude with incremental flash intensities (at least threefold increase at 10 and 30 cd s/m^2^) implying partial rescue of photoreceptor light response (Fig. 1d). In contrast to vehicle-treated mice, JQ1-treated rd10 mice showed significantly preserved inner retina (ON-bipolar and Muller cell) b-wave amplitudes (doubled) at all flash intensities. The integrity of inner retina is also reflected by preserved oscillatory potential (OP) response overlapping b-waves (Fig. 1c). These results agree with the morphometric data in Fig. 1b suggesting a protective effect of BET blockade on photoreceptor survival.

**Fig. 2:**
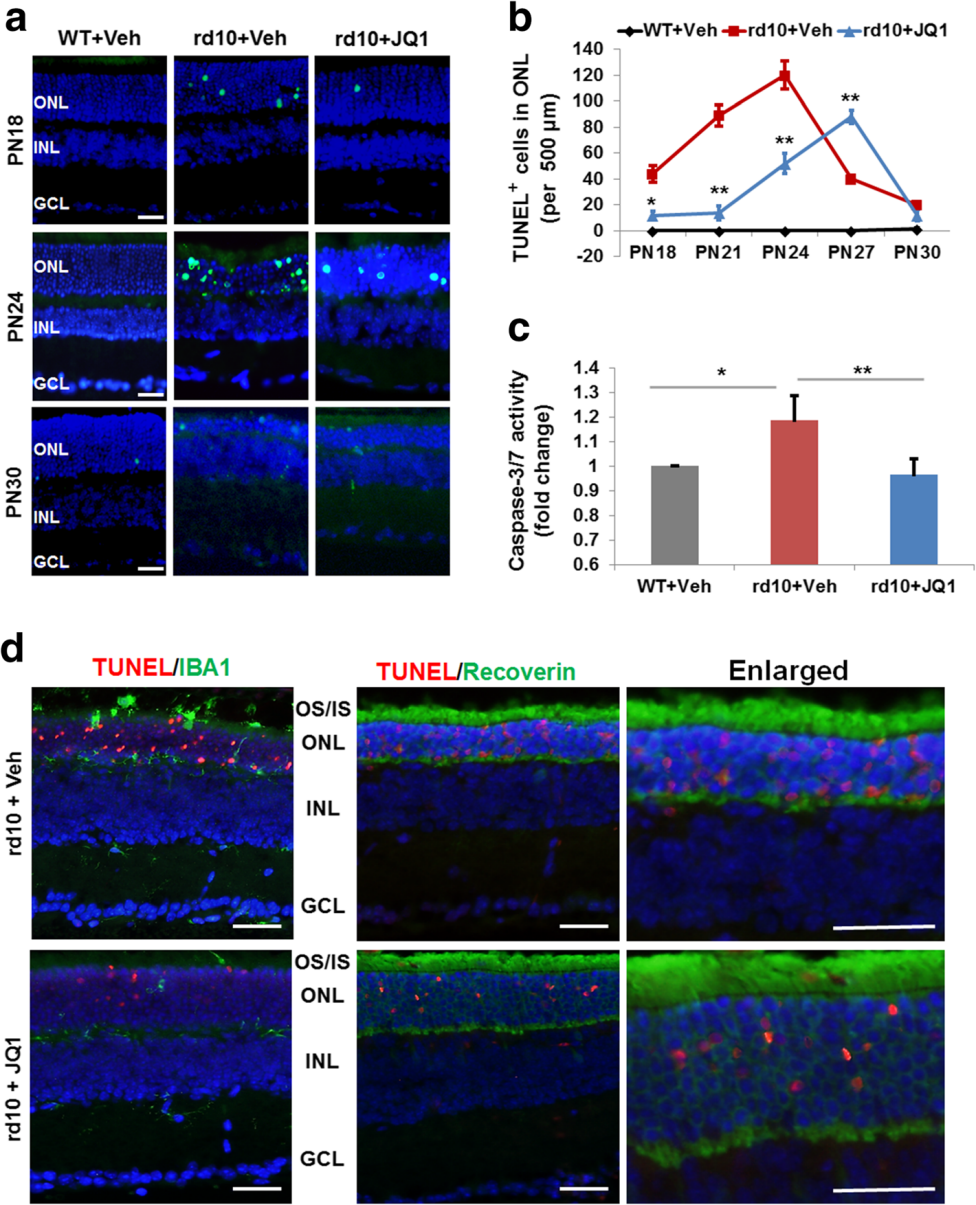
JQ1 treatment inhibits apoptosis in the rd10 mouse retina. Intravitreal injection of JQ1 (or vehicle) was performed at PN14, as described in Fig. 1. Eyeballs were collected at PN18, PN21, PN24, PN27, and PN30. Cryosections were prepared and used for TUNEL staining. **a** Representative TUNEL (*green*) images showing an inhibitory effect of JQ1 in rd10 retinas. *Scale bar* 50 μm. *Blue*: DAPI staining of nuclei. For images of PN21 and PN27, see Additional file 1: Figure S2. **b** Quantification of TUNEL-positive cells (per 500 μm ONL length): mean ± SEM, *n* = 6 mice; **P < 0.01, *P < 0.05 compared to rd10 vehicle control. **c** Caspase-3/7 activity assay showing an inhibitory effect of JQ1 on retinal cell apoptosis. For the assay, homogenates were prepared from retinas collected and pooled from six mice at PN24. **P < 0.01 compared to rd10 vehicle control, *n* = 3 independent assay experiments. **d** For TUNEL/IBA1 and TUNEL/Recoverin co-staining (PN24 retinas), a TMR red kit (Roche) was used so that TUNEL-positive nuclei appear red. The data show that TUNEL-positive nuclei do not overlap with IBA1-positive cells; instead, they are localized within recoverin-positive (photoreceptor) cells. *Scale bar* 50 μm

To further confirm the retinal protective effect of JQ1, we assessed apoptosis (Fig. 2, Additional file 1: Figure S2), either by TUNEL staining on retinal sections, or by caspase-3/7 activity assay using retinal homogenates. Consistent with previous reports [26, 27], TUNEL-positive cells in vehicle-treated rd10 retinas continuously increased from PN18 to PN24 and then decreased at later time points. In contrast, JQ1 treatment significantly reduced TUNEL-positive cells in ONL during PN21-PN24 and appeared to have shifted or delayed the time course of photoreceptor apoptosis compared to vehicle control (Fig. 2b). Accordantly, JQ1 significantly reduced retinal caspase-3/7 activity at PN24 (Fig. 2c). As shown in Fig. 2d, essentially all TUNEL-stained nuclei are found in recoverin (photoreceptor marker)-positive cells, indicating photoreceptor apoptosis characteristic of rd10 mice [26]. Moreover, the lack of overlap between TUNEL-stained nuclei and IBA1-positive cells also supports the conclusion that apoptosis occurred mainly in photoreceptor cells rather than in microglia. Taken together, our data indicate that blocking BET bromodomains in the rd10 retina via intravitreal injection of JQ1 preserves photoreceptor number and function and reduces (or delays) its apoptosis. The decrease of JQ1 protective effects at later time points likely stems from the fact that the JQ1/BET binding is reversible and dissociated JQ1 can diffuse out or decompose [13]. We used 0.1 mM JQ1 for intravitreal injection throughout the in vivo experiments because higher concentrations (0.5 and 2 mM) did not provide greater protective effect (Additional file 1: Figure S4).

### Blocking BETs with JQ1 mitigates microglial activation in the rd10 mouse retina

**Fig. 3:**
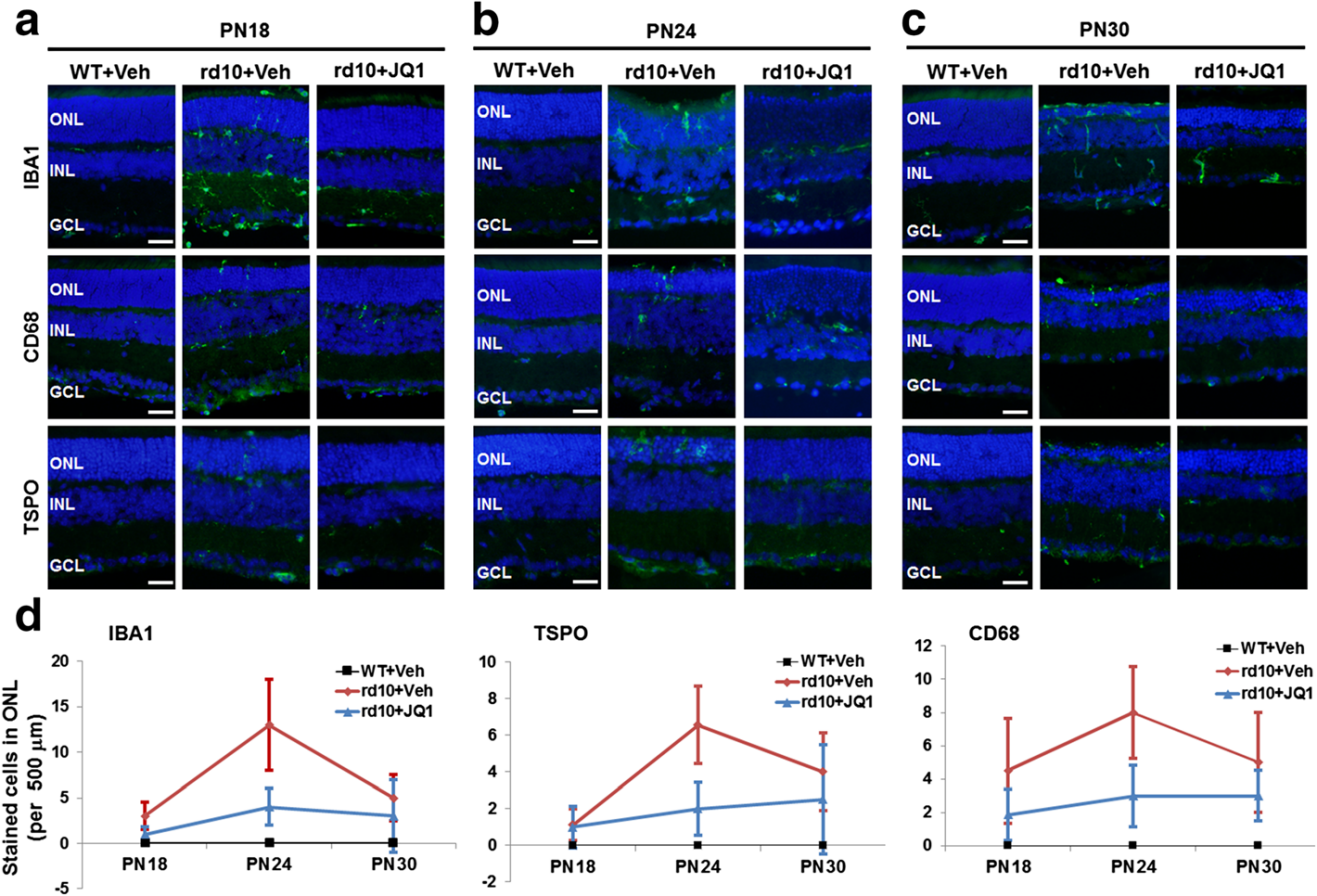
JQ1 treatment inhibits retinal microglial activation in the rd10 retina. Intravitreal injection of JQ1 (or vehicle) was performed at PN14, as described in Fig. 1. Eyeballs were collected at PN18, PN24, and PN30. Cryosections were used for immunostaining and fluorescence microscopy. **a**–**c** Representative immunostaining images of microglial markers. *Scale bar* 50 μm. *Blue* DAPI staining of nuclei. **d** Quantification: IBA1, CD68, or TSPO positive cells per 500 μm ONL length; mean ± SEM, *n* = 6 mice. Quantification of IBA1 staining at all five time points (PN18-PN30) is presented in Additional file 1: Figure S3

Recent studies showed that microglial activation plays an important role in retinal photoreceptor loss in rd10 mice [6, 7]. In parallel, an in vitro study found that JQ1 treatment inhibits lipopolysaccharide (LPS)-stimulated inflammation in the BV-2 microglial cell line [17]. However, it remains unknown whether blocking the BET family with JQ1 suppresses microglial activation in vivo in the degenerating rd10 mouse retina. To address this question, we performed immunostaining of microglial markers [7] IBA1, TSPO, and CD68 on retinal sections (Fig. 3a–c). At PN24, there was a dramatic increase of these microglial marker proteins in the photoreceptor region (ONL) compared to B6 controls (Fig. 3d), suggesting microglial proliferation and migration from inner layers [6, 7], both characteristic of microglial activation. JQ1 treatment substantially reduced cells positively stained for these marker proteins (Fig. 3d, Additional file 1: Figure S3), suggesting a decrease of activated retinal microglia.

**Fig. 4:**
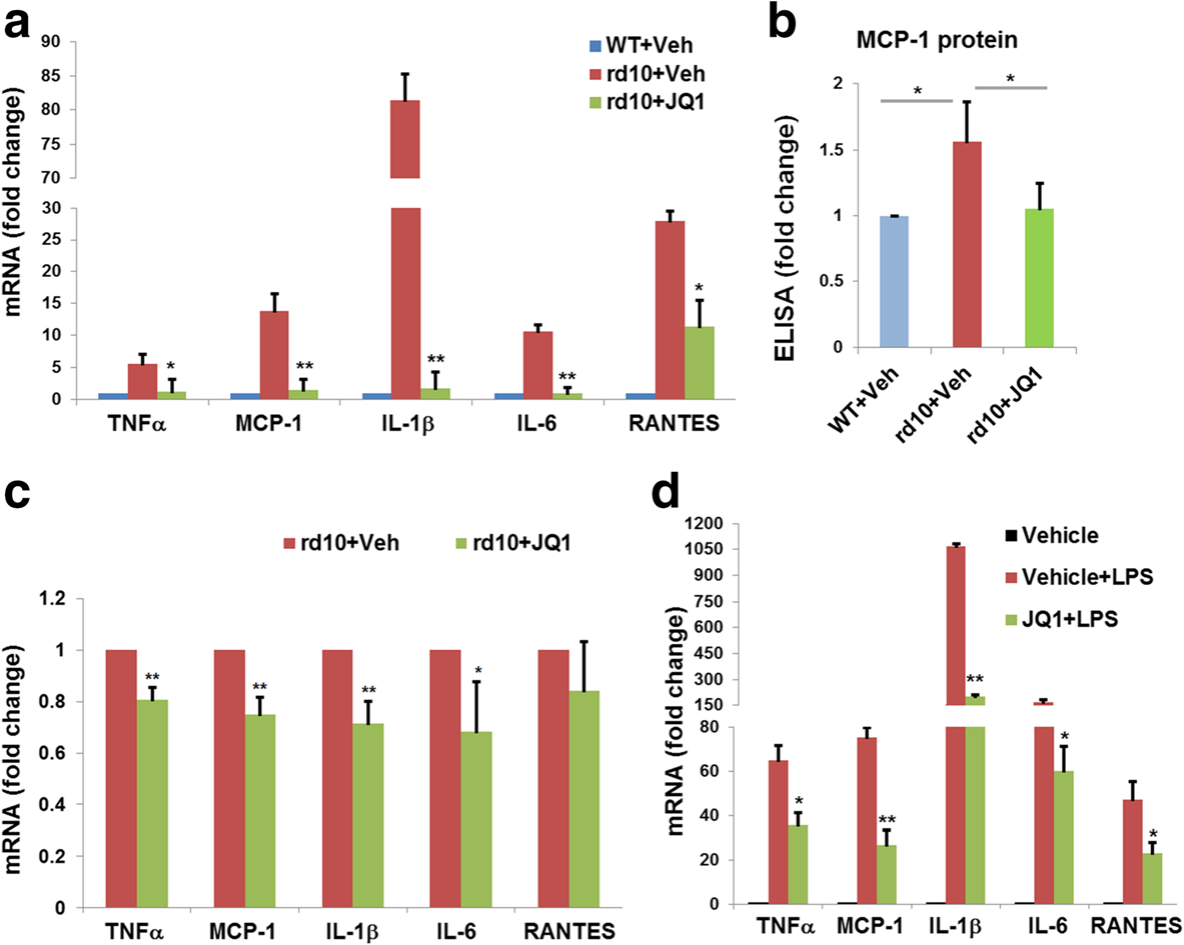
JQ1 treatment inhibits microglial expression of inflammatory cytokines in the rd10 retina. Intravitreal injection of JQ1 (or vehicle) was performed at PN14, as described in Fig. 1. Retinas were collected at PN24. **a** Homogenates were prepared from retinas (3 groups, total 12 mice) and used for qRT-PCR. Quantification: normalization to GAPDH mRNA; mean ± SEM; *n* = 3 independent homogenation/qRT-PCR experiments; *P < 0.05, **P < 0.01 compared to rd10 vehicle control. **b** Retinal homogenates were used for determination of MCP-1 protein levels by ELISA. Quantification: normalization to WT mice injected with vehicle; mean ± SEM; *n* = 3 experiments; *P < 0.05. **c** Retinal microglia were purified from dissociated rd10 retinas via flow sorting, as described in “Methods”. Sorting was performed five times using retinas collected from five groups of rd10 mice (four mice each). Sorted microglial cells (CD11b + CD45low) were then subjected to qRT-PCR. Quantification: normalization to GAPDH mRNA; mean ± SEM; *n* = 5; *P < 0.05, **P < 0.01, compared to rd10 vehicle control. **d** Microglial cells were isolated and purified from B6 mouse brains. Cells were pre-incubated with vehicle (DMSO) or JQ1 (0.5 μM) for 12 h, and then induced for inflammatory cytokine expression with LPS (1 μg/ml) for another 2 h prior to qRT-PCR assay. Quantification: normalization to GAPDH mRNA; mean ± SEM; *n* = 3 independent experiments; *P < 0.05, **P < 0.01 compared to vehicle + LPS

To determine the gross effect of JQ1 on retinal inflammation, we used retinal homogenates of rd10 mice treated by intravitreal injection of vehicle or JQ1. As shown in Fig. 4a, whereas inflammatory cytokine (TNFα, MCP-1, IL-1β, IL-6, and RANTES) mRNAs in the rd10 retina markedly increased compared to B6 controls, JQ1 treatment effectively reduced their expression to basal WT (B6) levels. This result was also supported by ELISA assay of MCP-1 protein levels (Fig. 4b).

We then assessed the effect of JQ1 specifically on retinal microglia inflammation, using retinal microglial cells directly purified (by sorting, Additional file 1: Figure S5) from rd10 mice treated with JQ1 or vehicle. We found that the expression of inflammatory cytokines was significantly (except RANTES) reduced in JQ1-treated retinal microglia compared to vehicle control (Fig. 4c). The manipulations during cell isolation and purification may have inevitably dampened the JQ1 effect on the expression of inflammatory cytokines.

To further confirm the anti-inflammatory effect of JQ1 specifically on microglia, we used primary microglial cell cultures, derived from whole mouse brain, which is an abundant source of microglial cells. We found that in response to LPS stimulation, inflammatory cytokines TNFα, MCP-1, IL-1β, IL-6, and RANTES were all dramatically upregulated, and JQ1 pre-incubation significantly reduced LPS-stimulated expression of all these cytokines (Fig. 4d).

These different lines of evidence collectively suggest a prominent role of the BET family in microglial activation in the degenerating retina of rd10 mice.

### Blocking BETs abrogates inflammation, proliferation, and migration of N9 microglial cells

**Fig. 5:**
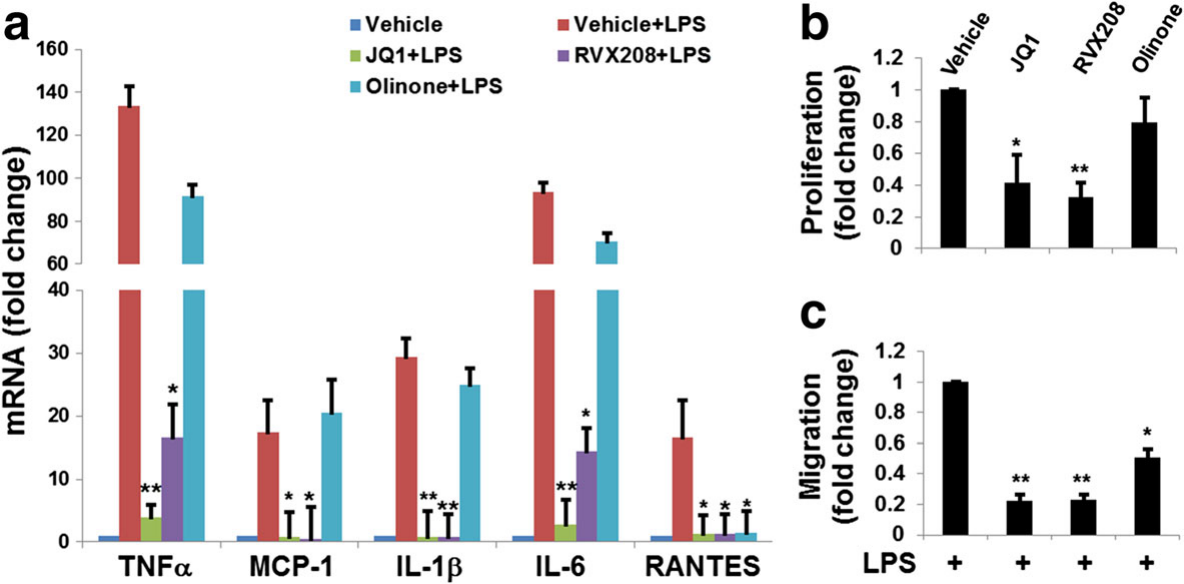
JQ1 pre-treatment inhibits N9 microglial cell inflammation, proliferation, and migration. N9 microglial cells were pre-incubated with vehicle (DMSO), JQ1 (0.5 μM), RVX208 (30 μM), or Olinone (30 μM) for 12 h followed by stimulation with LPS (1 μg/ml) for another 2 h, and then subjected to qRT-PCR (**a**) for determination of inflammatory cytokine expression. Proliferation assay (**b**, BrdU ELISA) and migration assay (**c**, Transwell) were performed after incubation for additional 24 h. Quantification: mean ± SEM; *n* = 3 experiments; *P < 0.05, **P < 0.01 compared to vehicle + LPS

To further analyze the role of BET proteins in microglial activation in a systematic manner, we used N9 cells, a commonly used microglial cell line originally provided by Dr. Paula Ricciardi-Castagnoli [23]. Using a cell line allowed us to perform genetic modifications, e.g., knockdown, and assess major pathogenic phenotypes of activated microglia, including inflammation, proliferation, migration, and elevated expression of inflammatory factors. As shown in Fig. 5a, while LPS potently stimulated the expression of inflammatory cytokines (TNFα, MCP-1, IL-1β, IL-6, and RANTES), JQ1 pre-treatment abolished this LPS-stimulated phenotype of activated microglia. Furthermore, JQ1 pre-treatment reduced N9 cell proliferation and migration by ~70% (Fig. 5b, c). We used a concentration of 0.5 μM JQ1 based on an N9 cell viability dose-response (Additional file 1: Figure S6).

Thus, these combined data from the N9 cell line are consistent with the results that blocking BET bromodomains with JQ1 effectively suppresses microglial activation in the rd10 retina (Figs. 3 and 4).

The BET inhibitors thus far developed are not selective within the BET family. JQ1 is highly specific to the BET family, but binds with similar affinities to both bromodomains (Brom1 and Brom2) of all family members [24]. After establishing the inhibitory effect of JQ1 on microglial activation, we further explored which bromodomain (Brom1 or Brom2) is the primary functional site responsible for the observed JQ1 effect using inhibitors specific to either Brom1 or Brom2. While Brom2 blocker RVX208 [28] abrogated major phenotypes of activated microglia (inflammation, proliferation, and migration), Brom1 blocker Olinone [29] effectively inhibited N9 cell migration, but not proliferation (Fig. 5b, c). Moreover, while RVX208 abolished the expression of all the tested inflammatory cytokines, Olinone blocked LPS-stimulated upregulation of RANTES, indicating functional activity of the drug, but it had no effect on the mRNA expression of TNFα, MCP-1, IL-1β, and IL-6 (Fig. 5a).

Taken together, these results demonstrate that BET epigenetic readers play an essential role in microglial activation, and that the two bromodomains may have differential functions in this pathogenic process.

### BET2 but not BET3 and BET4 is upregulated in the retina of rd10 mice compared to wild-type mice; BET2 but not BET4 knockdown effectively inhibits N9 microglial cell inflammation

**Fig. 6:**
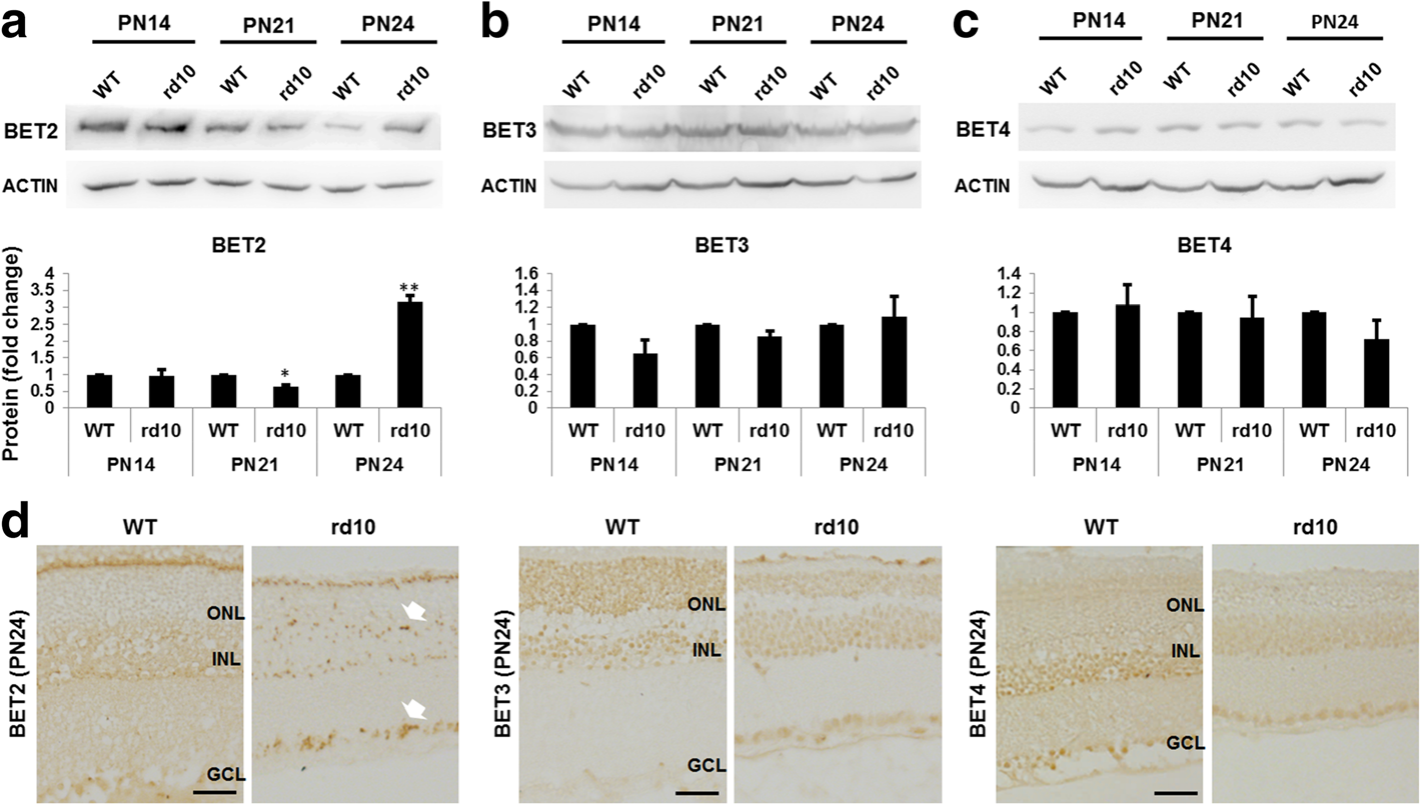
BET2 is upregulated in the rd10 mouse retina compared to B6 WT control. **a**–**c** Western blots of retinal homogenates collected from B6 or rd10 mice at PN14, P21, or PN24 (>6 mice at each time point). Quantification: normalization to β-actin and WT at each time point; mean ± SEM; *n* = 3 independent Western blot experiments; *P < 0.05, **P < 0.01 compared to WT control. **d** Immunostaining of BET2, BET3, and BET4 on paraffin-embedded retinal sections collected at PN24. *Arrow* points to *dots* of condensed staining. *Scale bar* 50 μm

As JQ1 is BET-specific but does not discriminate between BET family members, we analyzed which BET protein is the most likely target responsible for the observed JQ1 inhibitory effect on microglial activation. The BET family is comprised of BET2, BET3, and BET4. A fourth member, BRDT, is testis-specific [30] and thus irrelevant to the current study. The expression of BET family members in the retina has not been previously reported. We determined BET protein levels by Western blotting using retinal homogenates collected from rd10 and WT (B6) mice at different ages. Interestingly, BET2 protein levels dramatically decreased from PN14 to PN24 in B6 mouse retinas, but maintained in rd10 retinas and thus became significantly higher than in B6 mice at PN24 (Fig. 6a). In contrast, either BET3 or BET4 protein levels did not change over age and were equivalent between rd10 and B6 retinas at three age points (Fig. 6b, c). These results are consistent with a role of BET2 in retinal degeneration in rd10 mice. In addition, BET2 immuno-staining is evenly spread out on B6 sections, but shows a distinct pattern on rd10 sections at PN18-PN30, i.e., condensed dots in ONL and inner layers. In contrast, there is no obvious difference of BET3 or BET4 staining between B6 and rd10 retinal sections at all ages examined (Fig. 6d, Additional file 1: Figure S7). We thus focused on BET2 as the most relevant of the three BETs to microglial activation.

**Fig. 7:**
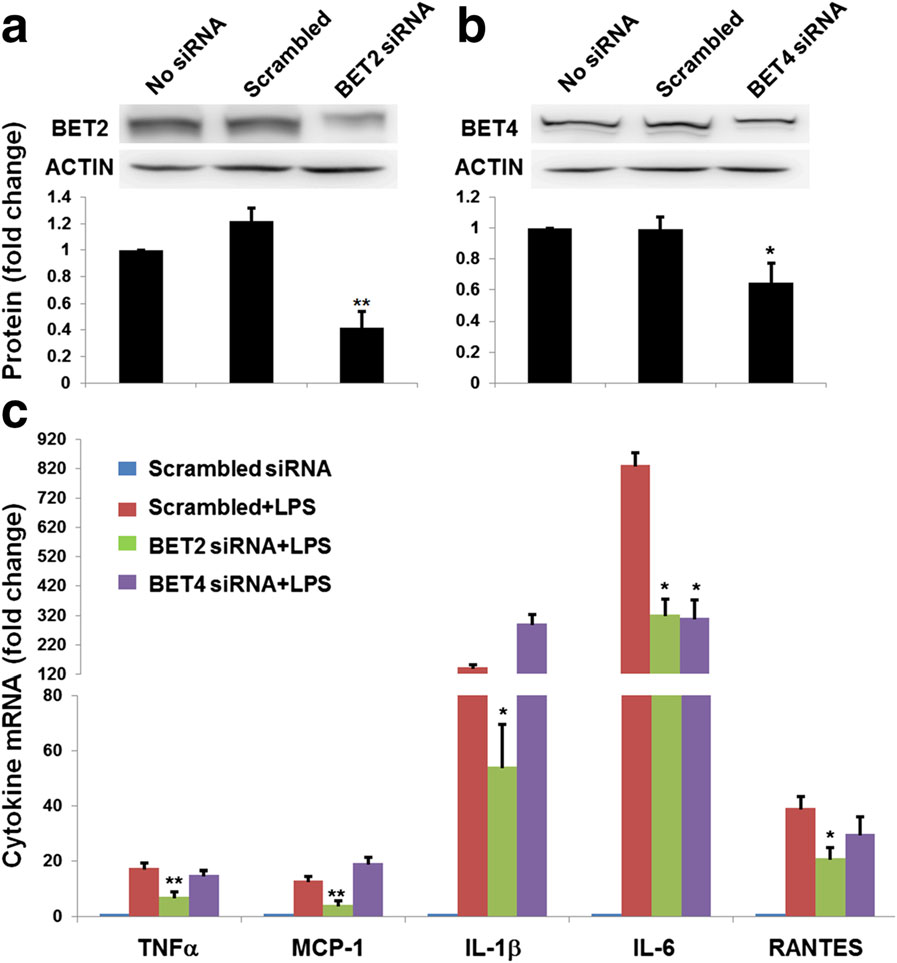
BET2 knockdown in N9 microglial cells inhibits inflammatory cytokine expression. N9 cells were infected with lentivirus expressing BET2 or BET4 siRNAs for 3 days and then GFP-positive (infected) cells were purified by flow sorting and cultured for 2–3 days. The cells were either used for Western blotting or stimulated with LPS (1 μg/ml) for 2 h and then subjected to qRT-PCR. **a**, **b** Western blots showing BET protein knockdown. Quantification: mean ± SEM; *n* = 3 experiments; *P < 0.05, **P < 0.01 compared to scrambled siRNA. **c** qRT-PCR determination of inflammatory cytokine mRNAs. Quantification: mean ± SEM; *n* = 3 experiments; *P < 0.05, **P < 0.01 compared to scrambled siRNA + LPS

We performed siRNA knockdown to further define the role of BET2 in microglial activation (Fig. 7). As BET4 was also implicated in previous studies as a pro-inflammatory regulator in macrophages [9, 15], we also knocked down BET4 in N9 cells. Efficient knockdown of both BET2 and BET4 by specific siRNAs was confirmed by Western blotting (Fig. 7a, b) as well as qRT-PCR (Additional file 1: Figure S8). We found that although BET2 knockdown inhibited LPS-induced expression of all tested inflammatory cytokine genes (TNFα, MCP-1, IL-1β, IL-6, and RANTES) by ~50%, BET4 knockdown did not produce a significant inhibition of expression of any cytokine except IL-6 (Fig. 7c).

## Discussion

Epigenetic modulation is becoming an attractive strategy for developing new therapeutics. In particular, the BET epigenetic “reader” family has recently garnered tremendous attention [10], primarily because of the discovery of highly specific designer inhibitors to these notoriously “undruggable” regulators [13], some of which quickly advanced to clinical trials [14]. The BET family has been recently identified as a prominent player in a growing list of pathological conditions, predominantly proliferative and inflammatory diseases [10]. However, whether BET proteins are involved in retinal degenerative diseases is not known. In this study, we made three main findings. First, blockade of bromodomains of the BET family with JQ1 preserves photoreceptor number and retinal function in the RP model of rd10 mice. Second, JQ1 treatment suppresses microglial activation in vivo in the rd10 retina and in vitro under pathogenic stimulation. Third, BET2 is likely a determinant BET family member and Brom2 appears to be the primary functional domain in the activation of N9 microglial cells. Inasmuch as microglial activation plays a crucial role in photoreceptor degeneration, as recently demonstrated in rd10 mice [6, 7], our results suggest that epigenetic interference targeting BET proteins (or bromodomains) may open a new avenue to protect photoreceptors via effective suppression of microglial activation.

Our findings are novel because we provide the first evidence for rescue of photoreceptors and inner retina (see b-wave, Fig. 1) in an inherited retinal degeneration model by disrupting BET epigenetic readers. Since the serendipitous discovery of the first-in-class BET inhibitor, JQ1 [13], and the ensuing development of various analogs [14], studying BET functions in diseases has become feasible [10]. Most of the recent breakthroughs surrounding BETs occurred in cancers, inflammatory diseases, and/or immunological disorders, supporting a key role of this family in pathogenesis [10, 14]. However, there are very few reports on BETs in the central nervous system. In fact, to the best of our knowledge, there is no publication reporting BET functions specifically in retinal degeneration. Two recent brain studies identified BET4-mediated transcriptional activation during memory formation [31] and cocaine-induced neuronal plasticity [32]. Another relevant study showed an inhibitory effect of JQ1 on human umbilical vein endothelial cell proliferation, migration, and tube formation [33]. While in this new report, JQ1 was found to inhibit neovascularization in an oxygen-induced retinopathy mouse model, its effect on retinal degeneration was not investigated. Therefore, almost nothing is known about BET regulation in retinal degeneration, underscoring the urgency of research in this area.

While our data show an in vivo role of BETs in microglial activation during retinal degeneration, our finding is also supported by relevant recent reports. One study using the BV-2 microglial cell line and RNA sequencing indicated JQ1 inhibited LPS-stimulated expression of inflammation- and immunity-related genes [17]. Other two studies demonstrated suppression of inflammatory gene expression by blocking the BET family in LPS-stimulated macrophages [9, 15], which are monocyte-derived immune cells with similarities to microglia [16]. Most recently, anti-inflammatory effect of BET inhibition was observed in the mouse brain [34]. Our study is distinct from these reports in that our data provide in vivo evidence for the suppression of microglial activation via BET inhibition specifically in the retina undergoing neurodegenerative pathology. We observed dramatically reduced microglial infiltration into the ONL and subretinal regions in JQ1-treated rd10 retinas versus vehicle control-treated retinas. Moreover, gene expression determination using microglia directly isolated from rd10 retinas confirmed that JQ1 treatment reduces microglial inflammation in the retina. Significantly, we observed that JQ1 treatment preserves photoreceptors in the rd10 model. Based on recent reports that microglial activation potentiates photoreceptor demise in rd10 mice [6, 7], we infer JQ1 protects photoreceptors in large part by suppressing microglial activation. In addition, we found JQ1 also reduces apoptosis in the rd10 photoreceptor layer. Our data cannot distinguish whether this was a direct effect on the apoptotic program in photoreceptors or a secondary effect via inhibition of microglial activation which promotes photoreceptor apoptosis [6]. Since it is not technically feasible to homogenously isolate and culture retinal photoreceptors, it will require future investigation in photoreceptor-specific BET knockout mice to definitively determine whether BETs regulate the apoptotic program directly in photoreceptors. However, the proposition of direct BET regulation in photoreceptor cells in this context is undermined by little positive staining of the BETs (if any above non-specific background) in the ONL photoreceptor nuclei. Nevertheless, our results support a promising strategy to protect photoreceptors in RP via pharmacological inhibition of the BET family, a distinct group of epigenetic readers.

It is worthnoting that despite a reported short half life (~1 h) of JQ1 after intraperitoneal injection into mice [13], in our experiments, *intravitreally* delivered JQ1 produced photoreceptor protection even 10 days after injection. There are at least two plausible explanations for this: (1) The drug delivered into the eye, an isolated organ, may not immediately enter the circulation thus evading quick metabolic degradation. (2) Even if JQ1 binds BET proteins only at early times, consequential changes in gene expression and downstream signaling could have a lasting effect. In future investigations, further prolonged therapeutic benefits may be achieved by using a JQ1 derivative with improved bioavailability (or half life). Moreover, since higher doses of injected JQ1 did not significantly improve its therapeutic effect (Additional file 1: Figure S4), a more sophisticated delivery method should be applied, e.g., using nanoparticles to extend drug release time or an osmotic pump to provide continued release.

While a recent study by Jung et al*.* showed a prominent role of JQ1 in suppressing LPS-induced BV-2 cell inflammatory gene expression [17], it is interesting to note distinct outcomes of our study using N9 cells, another commonly used microglial cell line [35]. We found that blocking BET activity with JQ1 effectively abrogated LPS-stimulated upregulation of TNFα, IL-1β, and MCP-1 (CCL2). Elevation of TNFα and IL-1β is a hallmark of neuroinflammation, which is a critical etiology in neurodegenerative diseases [35]. MCP-1, a chemoattractant and an inflammatory cytokine, plays a crucial role in microglial migration/infiltration and neuroinflammatory disorders [8]. Using BV-2 cells, Jung et al*.* also observed JQ1 inhibition of LPS-induced transcription of IL-1β and MCP-1, but not TNFα [17]. Moreover, whereas our data of N9 cells show a JQ1 inhibitory effect on the expression of IL-6 and RANTES, two important inflammatory factors associated with microglial activation, they are absent from the list of JQ1-downregulated genes in BV-2 cells [17]. Supporting our results from the N9 cell line, we also observed JQ1-effected downregulation of the foregoing group of inflammatory cytokines in primary mouse brain microglial cells as well as primary microglia isolated and purified from rd10 mouse retinas. Together with our unique data on JQ1 inhibition of N9 cell proliferation and migration (Fig. 5b, c), our results demonstrate a previously uncharacterized broad potency of BET inhibition in blocking microglial activation. As BV-2 is a rat microglial cell line and N9 is derived from mouse brain microglia [23, 35], the discrepancy between our study and the previous report by Jung et al. [17] may arise from different origins of these two microglial cell lines.

Another distinction between the two studies is that in the previous report [17], it remained unknown as to which BET member or bromodomain plays a predominant role in microglial activation. Our siRNA experiments suggest that BET2 is the key regulator in microglial activation. There are several lines of evidence supporting this conclusion. First, BET2 knockdown by siRNA abolished LPS-induced expression of all tested inflammatory cytokines whereas BET4 knockdown did not produce a prominent effect. Second, BET2 protein levels in the retina (determined by Western blotting) were markedly increased at PN24 in rd10 mice compared to B6 mice, an age coinciding with the peak time of retinal microglial activation and photoreceptor degeneration in rd10 mice [6]. Third, consistent with the Western blot result, immuno-histochemistry on PN24 rd10 retinal sections revealed dots of condensed BET2 staining in the ONL and INL regions, a pattern distinct from that on B6 retinal sections. In contrast, neither BET3 nor BET4 staining shows a difference between rd10 and B6 retinas. Last, BET2 has been shown to be essential in LPS-induced inflammatory cytokine production in bone marrow-derived macrophages [15]. In this previous study, siRNA knockdown of BET2 or BET4 suppressed the expression of major inflammatory cytokines, including TNFα, IL-6, and MCP-1, and both BET2 and BET4 were found to associate with promoters of those genes. However, in our study using N9 microglial cells, knockdown of BET4 did not inhibit LPS-stimulated expression of TNFα, IL-1β, MCP-1, and RANTES. This difference in the two studies highlights the cell type and context dependence of BET regulation, which has been repeatedly observed in recent reports (see review [12]). On the other hand, knockdown of BET4 effectively blocked LPS stimulation of IL-6 transcription but not of other tested cytokines (Fig. 7c). This result suggests differential BET2 and BET4 regulations of inflammatory cytokine genes in microglia. We cannot rule out the possibility that BET4 may regulate other inflammatory cytokine genes not tested in the current study.

As JQ1 is a pan-specific inhibitor that blocks both Brom1s and Brom2s in all BET members, we also explored which bromodomain is the likely functional site of the observed JQ1 effects, using two inhibitors specific to either Brom1s or Brom2s in all BETs. Our data suggest that Brom2 may play a dominant role in BET-directed microglial activation. To our knowledge, differential roles of the two BET bromodomains in inflammatory gene expression have not been previously addressed. To determine definitively whether Brom2 in BET2 is the primary functional domain responsible for retinal microglial activation, future investigations should use microglia-specific BET knockout or bromodomain-inactivating mutant mice. Nevertheless, our results contribute new insights into the differential roles of BET family members and their bromodomains in microglial inflammatory responses. This progress is significant in regard to future development of RP-preventing therapeutics with maximal efficacy and minimal side effects, which may be achieved via precise BET targeting. In fact, development of BET protein- or bromodomain-selective inhibitors represents an active research area [24].

As supported by recent discoveries on BET epigenetic mechanisms, BET protein(s) may play a “master” regulator role during microglial activation. Genome-wide investigations reveal that a specific cell state is defined by the combination of only a small number of transcription factors and super-enhancers [11, 36, 37]. In response to pathogenic cues, transcription factors and super-enhancers re-assemble at and activate the expression of a select group of genes which act in concert to drive cell state transformation [11, 38, 39]. BET proteins play a critical role by coupling this transcription-activating assembly to RNA polymerase II [12]. When BET is displaced from epigenetic marks (acetylated-lysines) by a bromodomain blocker such as JQ1, the assembly collapses [12]. Thus, BET proteins and/or bromodomains provide sensitive pharmacological targets for interventions. This mechanism may underlie the profound inhibitory effect of JQ1 on microglial activation. Our future studies on BET-associated transcription factors and super-enhancers are expected to elucidate this possible scenario in retinal microglial activation.

## Conclusions

We have identified a prominent role of the BET epigenetic reader family in photoreceptor degeneration and retinal microglial activation in the rd10 RP model. Our study advocates a BET-targeted novel strategy for effective treatment of RP. The recent development of BET inhibitors represents a new chapter in “epigenetic therapy” because they are the first successful pharmacological interference with the class of epigenetic “readers” [14]. Only a few years after the first description of BET-specific inhibitors [9, 13], several drugs are already in clinical trials with encouraging results [10, 40]. Intriguingly, recent studies indicate that blockade of BETs, seemingly general transcriptional co-activators, does not suppress genes globally, but rather, suppresses only over-active genes, which are often pathogenic [10–12]. This specificity of BET-governed regulations is a solid foundation for future translation of a BET-targeted therapeutic paradigm. As microglial activation, or pathogenic cell state transformation, is a hallmark of multiple neuro-degenerative diseases [8], our study may have an important impact beyond the rd10 RP model and retinal degenerative diseases.

## Additional file

Additional file 1: Supplemental materials. (DOCX 12721 kb)

## Abbreviations

BET: Bromodomain and extraterminal domain proteins
CD68: Cluster of differentiation 68/macrosialin
LPS: Lipopolysaccharides
ONL: Outer nuclear layer
TUNEL: Terminal deoxynucleotidyl transferase dUTP nick end labeling
