# Supplementary material for: Photoreceptor protection via blockade of BET epigenetic readers in a murine model of inherited retinal degeneration
Source: J Neuroinflammation. 2017 Jan 19;14:14. doi: 10.1186/s12974-016-0775-4 (PMC5248448; doi:10.1186/s12974-016-0775-4)
Supplement: Additional file 1: — Supplemental materials. (DOCX 12721 kb) [file 12974_2016_775_MOESM1_ESM.docx]

**Photoreceptor protection via blockade of BET epigenetic readers in a murine model of inherited retinal degeneration**

Lei Zhao^1^, Jun Li^1,2,3^, Yingmei Fu^1,4^, Mengxue Zhang^1^, Bowen Wang^1^, Jonathan Ouellette^5^, Pawan K. Shahi^6^, Bikash Pattnaik^6,8^, Jyoti J. Watters^5^, Wai T. Wong^7^, and Lian-Wang Guo^1,8^

^1^Department of Surgery, Wisconsin Institute for Medical Research, University of Wisconsin School of Medicine and Public Health, Madison, WI 53705, USA

^2^Department of Ophthalmology, the First Hospital of China Medical University, Shenyang, 110001, PR China

^3^Department of Ophthalmology, the 3rd People’s Hospital of Dalian, Dalian, 116033, PR China

^4^Shanghai Key Laboratory of Psychotic Disorders, Shanghai Mental Health Center, Shanghai Jiao Tong University School of Medicine, 600 Wanping Nan Road, Shanghai, 200030, PR China.

^5^Department of Comparative Biosciences, University of Wisconsin, Madison, WI 53706, USA

^6^Department of Pediatrics, Department of Ophthalmology and Visual Sciences, University of Wisconsin, Madison, Wisconsin, USA

^7^Unit on Neuron-Glia Interactions in Retinal Disease, National Eye institute, National Institutes of Health, Bethesda, MD, USA

^8^McPherson Eye Research Institute, University of Wisconsin, Madison, WI 53705, USA

Author email addresses: [zhaol@surgery.wisc.edu](mailto:zhaol@surgery.wisc.edu); [lij@surgery.wisc.edu](mailto:lij@surgery.wisc.edu); [fu@surgery.wisc.edu](mailto:fu@surgery.wisc.edu); [zhangm@surgery.wisc.edu](mailto:zhangm@surgery.wisc.edu); [wangb@surgery.wisc.edu](mailto:wangb@surgery.wisc.edu); [jouellette@wisc.edu](mailto:jouellette@wisc.edu); pshahi@wisc.edu; [pattnaik@wisc.edu](mailto:pattnaik@wisc.edu); [jjwatters@wisc.edu](mailto:jyoti.watters@wisc.edu); [wongw@nei.nih.gov](mailto:wongw@nei.nih.gov); [guo@surgery.wisc.edu](mailto:guo@surgery.wisc.edu)

Running Title: ***Role of BET epigenetic readers in retinal degeneration***

***Correspondence:***

Lian-Wang Guo, PhD

Assistant Professor

Department of Surgery

University of Wisconsin School of Medicine and Public Health

5151 Wisconsin Institute for Medical Research

1111 Highland Ave.

Madison, WI 53705, USA

Fax: 608 262 3330

Tel: 608 262 6269

E-mail: [guo@surgery.wisc.edu](mailto:guo@surgery.wisc.edu)

**Keywords:** Bromodomain and extraterminal domain (BET) proteins, epigenetic readers, retinal degeneration, microglial activation, JQ1

**Supplemental Materials**

**Table S1.** Primers used for quantitative real-time PCR (qRT-PCR).

| Gene Amplified | Forward | Reverse | Size (bp) |
| --- | --- | --- | --- |
| *Brd2* | TACTGGGCTGCCTCAGAATG | CCAGTGTCTGTGCCATTAGGA | 106 |
| *Brd3* | AAATGCAGGTTCCCAACAAGTG | CGGCAATGACGGGTGTCT | 101 |
| *Brd4* | CCCTGAAGCCATCTACACTACGA | ACCAGCAATCACGTCAACTTT | 101 |
| *IL1β* | GCAACTGTTCCTGAACTCAACT | ATCTTTTGGGGTCCGTCAACT | 89 |
| *IL6* | TCCAGTTGCCTTCTTGGGAC | GTACTCCAGAAGACCAGAGG | 331 |
| *Mcp1* | CCCACTCACCTGCTGCTACT | TCTGGACCCATTCCTTCTTG | 164 |
| RANTES | TGCCCTCACCATCATCCTCACT | GGCGGTTCCTTCGAGTGACA | 194 |
| *Tnfα* | CGCGACGTGGAACTGGCAGAA | GTGGTTTGCTACGACGTGGGCT | 276 |

**Supplemental Figures**


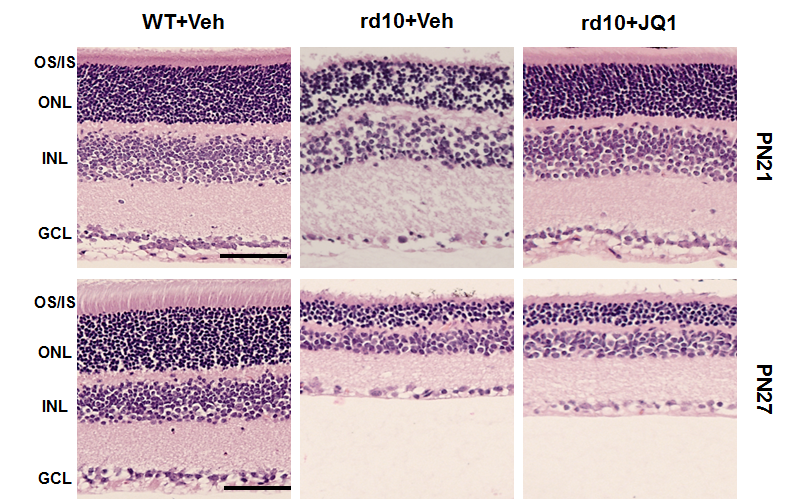


**Figure S1. *Images of H&E-stained retinal sections collected at PN21 and PN27***

Intravitreal injection of JQ1 (or vehicle) was performed at PN14, as described in Figure 1. At indicated time points, mice were sacrificed, and retinal cryo-sections were prepared and H&E stained. Images of more time points and quantification data are presented in Figure 1. Scale bar: 50 μm.


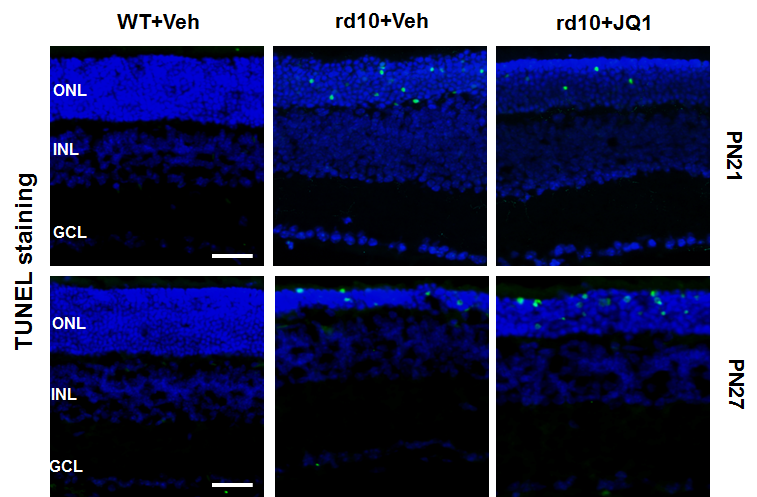


**Figure S2. *Images of TUNEL-stained retinal sections collected at PN21 and PN27***

Intravitreal injection of JQ1 (or vehicle) was performed at PN14, as described in Figure 1. At indicated time points, mice were sacrificed, and retinal cryo-sections were prepared for TUNEL staining. Images of more time points and quantification data are presented in Figure 2. Scale bar: 50 μm.


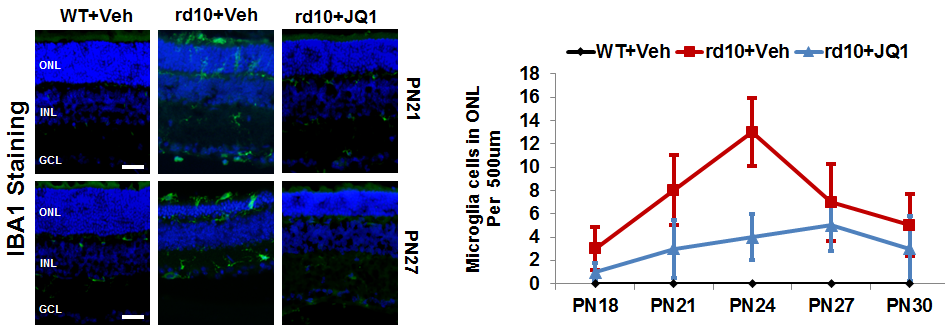


**Figure S3. *IBA1 staining on retinal sections collected at all time points (PN18-PN30)***

Intravitreal injection of JQ1 (or vehicle) was performed at PN14, as described in Figure 1. At indicated time points, mice were sacrificed, and retinal cryo-sections were prepared for IBA1 staining. Left panel: Representative immunostaining images of IBA1 at PN21 and PN27. Scale bar: 50 μm. Images of other time points are presented in Figure 3. Right panel: Quantification, IBA1-positive cells per 500 μm ONL length, mean ± SEM, n = 6 mice.


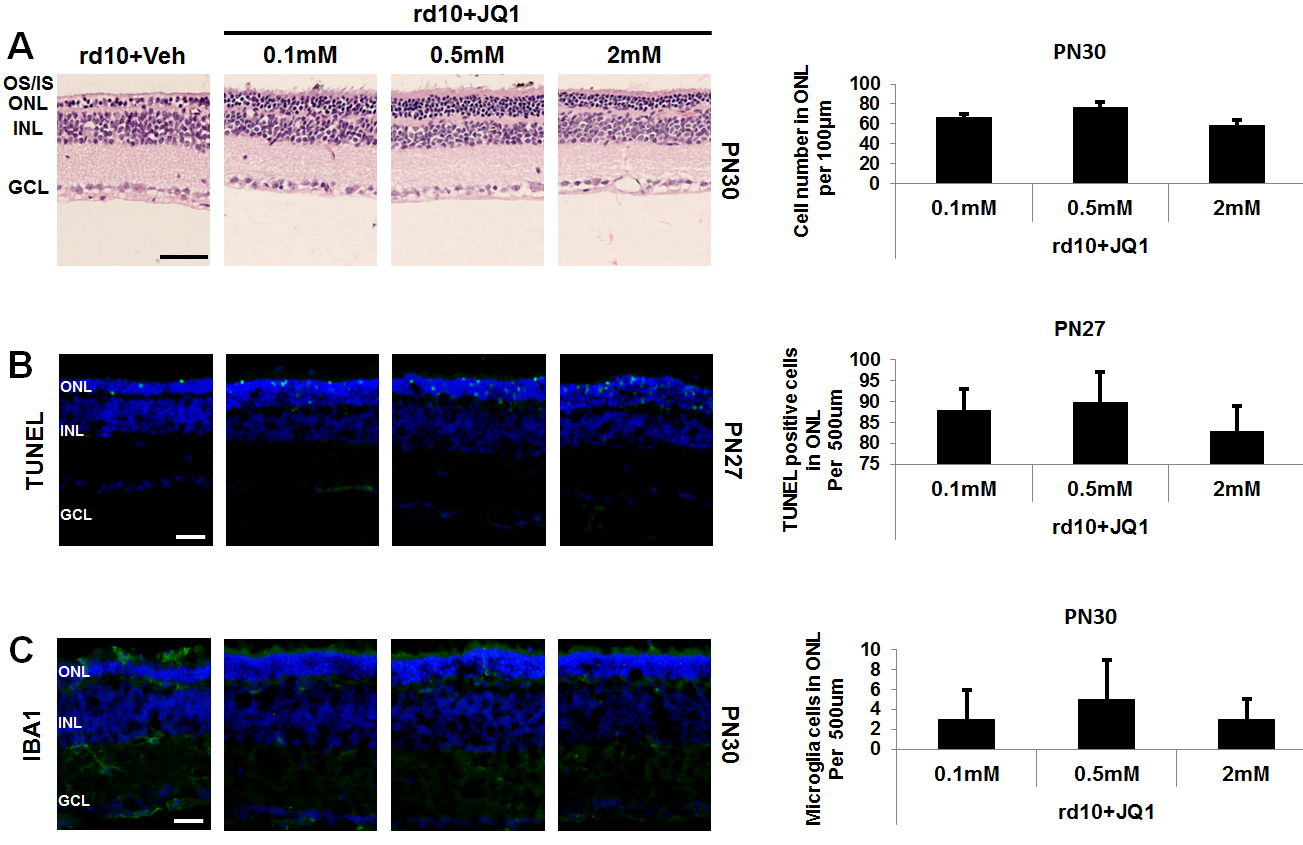


**Figure S4. *Three doses of JQ1 produce no difference in treatment effect***

Intravitreal injection of JQ1 (or vehicle) was performed at PN14, as described in Figure 1. At indicated time points (PN27 or PN30), mice were sacrificed, and retinal sections were prepared for H&E, TUNEL, or IBA1 staining. **A-C.** Representative staining images. Scale bar: 50 μm. Quantification is presented on the right, mean ± SEM, n = 5 mice. The data show that three different doses of JQ1 (0.1, 0.5, and 2 mM) did not produce significantly different effects. Thus the dose of 0.1 mM was used throughout the intravitreal injection experiments.


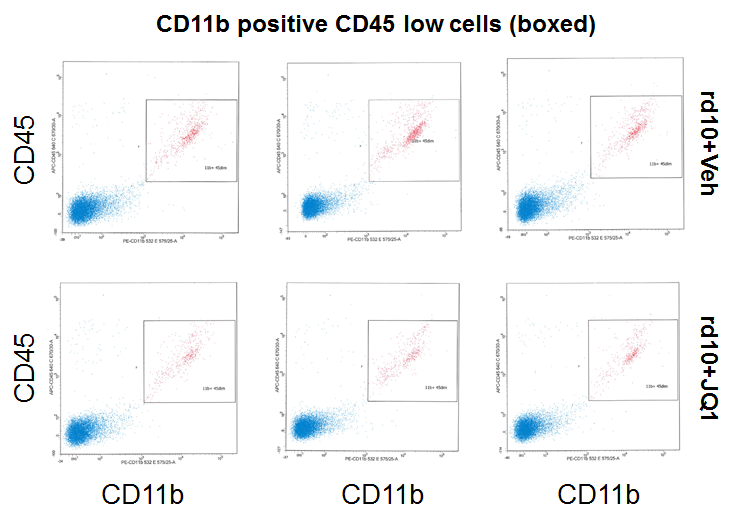


**Figure S5. *Purification of retinal microglial cells by flow sorting***

Intravitreal injection of vehicle and JQ1 was performed at PN14, as described in Figure 1. Retinas were collected from rd10 mice at PN24. Dissociation of retinal cells and purification of retinal microglial cells by flow sorting were performed as described in Methods. Shown are representative FACS plots. Blue: DAPI-stained cells, representing living cells; red: CD11b^+^/CD45^low^, defined as microglial cells.


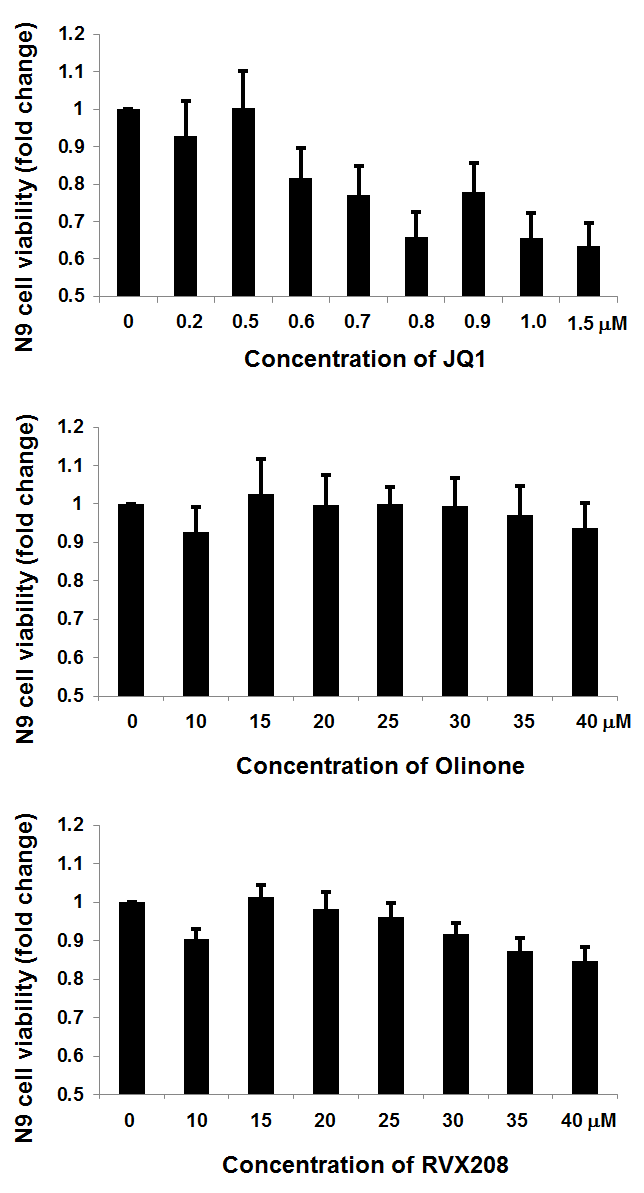


**Figure S6. *Response of N9 microglial cell viability to JQ1, RVX208, and Olinone concentrations***

In order to identify appropriate BET inhibitor concentrations for in vitro experiments, N9 microglial cells were pre-incubated with vehicle (DMSO), JQ1, RVX208, or Olinone for 12h, and then subjected to CellTiterGlo viability assay. Quantification: mean ± SEM; n = 3 experiments. We chose 0.5 μM JQ1, 30 μM RVX208, 30 μM Olinone for in vitro experiments throughout as these concentrations represent the “maximal” doses without affecting normal cell viability.


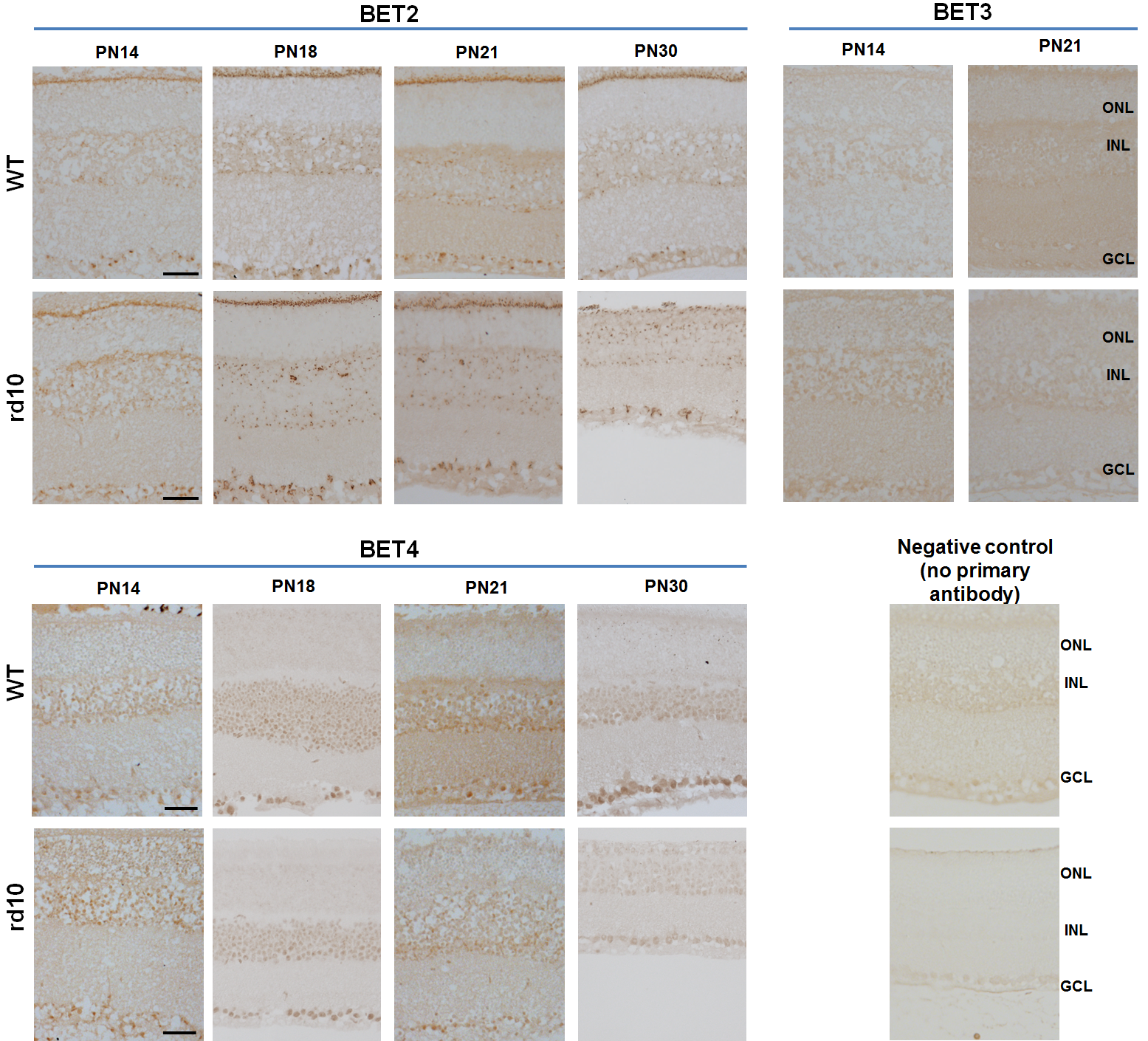


**Figure S7. *BET protein staining on retinal sections at PN14 and PN21***

As the same set of experiments, retinal paraffin section preparation and immunostaining of BET2, BET3, and BET4 were performed as described for Figure 6D where only PN24 sections are shown. “Negative control” refers to staining background without using a primary antibody. Scale bar: 50 μm.


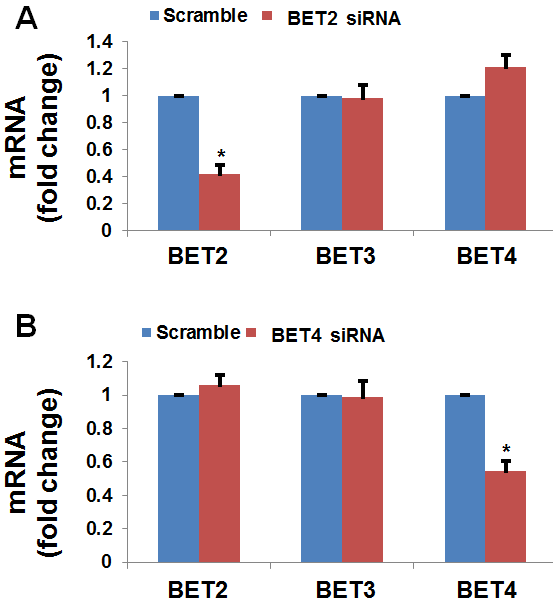


**Figure S8. *Specific knockdown using BET2 and BET4 siRNAs***

N9 cells were infected with lentivirus expressing BET2 (A) or BET4 siRNAs (B) for 3 days and then GFP-positive (infected) cells were purified by flow sorting and cultured for 2-3 days, as described for Figure 7. The cells were then subjected to qRT-PCR to determine mRNA levels of BET2, BET3, and BET4. Quantification: mean ± SEM; n = 3 experiments; *P < 0.05 compared to scrambled siRNA.
